# The use of rapid diagnostic tests for chronic Chagas disease: An expert meeting report

**DOI:** 10.1371/journal.pntd.0012340

**Published:** 2024-08-08

**Authors:** Freddy Perez, Debbie Vermeij, Roberto Salvatella, Luis Gerardo Castellanos, Andrea Silvestre de Sousa

**Affiliations:** 1 Communicable Diseases Prevention, Control, and Elimination Department, Pan American Health Organization, Washington, DC, United States of America; 2 Federal University of Health Sciences of Porto Alegre (UFCSPA), Porto Alegre, Rio Grande do Sul, Brazil; 3 Evandro Chagas National Institute of Infectious Diseases, Oswaldo Cruz Foundation, Rio de Janeiro, Brazil; 4 Department of Internal Medicine, School of Medicine, Federal University of Rio de Janeiro, Rio de Janeiro, Brazil; US Food and Drug Administration, UNITED STATES OF AMERICA

## Abstract

Chagas disease, caused by *Trypanosoma cruzi*, affects millions of people globally and is associated with significant underdiagnosis and undertreatment. Current diagnostic algorithms face challenges in remote regions. We aimed to review the potential of rapid diagnostic tests (RDTs) for screening or diagnosing chronic Chagas disease in endemic areas. An expert panel representing scientific and academic institutions from the Americas convened with the aim of discussing the use of RDTs. The study employed the nominal group technique, gathering insights from diverse experts during a 3-day meeting. Panel discussions covered RDT application, research protocols, and regulatory mechanisms. The results indicate that RDTs play a crucial role in surveillance and screening, although limitations in sensitivity and specificity exist. The expert group recommends standardized protocols, emphasizes the importance of cost-effectiveness assessments, and highlights the need to consider geographic validation. Despite these challenges, RDTs present a promising avenue for improving Chagas disease diagnosis in resource-limited settings. Future research and a collaborative approach are deemed essential for effective implementation.

## Author summary

Chagas disease, caused by the *Trypanosoma cruzi* parasite, affects approximately 7 million people globally, with potential cardiac or digestive complications in 30% of cases. Underdiagnosis and undertreatment in Latin America result from its often-silent progression, emphasizing the need for early and accurate diagnosis. A meeting of representatives from scientific and academic institutions discussed the use of rapid diagnostic tests (RDTs) for chronic Chagas disease covering RDT application, research protocols, and regulatory mechanisms, emphasizing the important role of this tool in rapidly detecting *T*. *cruzi*-specific antibodies. RDTs offer breakthroughs for remote areas. Currently recommended for screening, experts suggest evaluating their expanded use for definitive individual diagnosis, emphasizing their portability, minimal blood requirement, and rapid results. Challenges, including variability in RDT sensitivity and specificity, call for further research and evidence-based recommendations. The expert group highlights the need for standardized diagnostic protocols, reviewing regulatory aspects, and advocating for updates to diagnostic lists and improved supply management. In conclusion, the expert meeting provided a roadmap for optimizing RDTs in chronic Chagas disease diagnosis, balancing potential benefits with existing challenges. The proposed generic protocol aims to guide multicenter studies, positioning RDTs as valuable tools in addressing this public health issue across the Americas.

## Introduction

The World Health Organization (WHO) estimates that 7 million individuals worldwide are infected with *Trypanosoma cruzi (T*. *cruzi)*, causing Chagas disease; with 30% at risk of cardiac or digestive pathologies [1,2]. Despite a high disease burden, predominantly in Latin America, less than 10% of *T*. *cruzi* carriers are diagnosed, and only 1% receive treatment [3]. The silent nature of the disease contributes to underdiagnosis; up to 70% of infected individuals may not develop any symptoms nor specific organ damage. Early, accurate diagnosis is crucial for effective treatment and the implementation of clinical management, control, and elimination measures.

In line with international recommendations from the Pan American Health Organization (PAHO) [4], the diagnosis of *T*. *cruzi* infection in the chronic phase of Chagas disease is made using serological tests. WHO and PAHO strongly recommend the use of at least 2 serological tests representing different methodological principles or antigens: one with high sensitivity (for instance, immunoenzymatic test referred to as the enzyme-linked immunosorbent assay) and one with high specificity (for instance, indirect hemagglutination) [4,5]. In cases of ambiguous or discordant results, a third serological technique should be used (for instance, indirect immunofluorescence) [6]. Despite its effectiveness, this algorithm requires time, laboratory infrastructure, and expertise often unavailable in many rural and hard-to-reach regions, resulting in the underdiagnosis and underreporting of infected individuals.

Latin America’s complex health systems, which are set apart by the presence of diverse health subsystems, would benefit from the validation of suitable diagnostic tools for implementation in both highly complex facilities and primary care centers. Tools such as indirect chemiluminescence immunoassays (CLIAs), electrochemiluminescence immunoassays (ECLIAs), and chemiluminescence microparticle immunoassays (CMIAs) have been adopted by clinical laboratories at public healthcare facilities in major cities where fast, efficient, automated processing of a large number of samples is necessary [7]. In some Latin American countries, laboratories have independently begun implementing these tools to detect chronic *T*. *cruzi* infection, despite limited data on their diagnostic performance, using ad hoc algorithms depending on the jurisdiction involved. Before these techniques can be included in diagnostic algorithms recommended internationally by the PAHO, evidence obtained from diagnostic test evaluation studies should be analyzed to generate recommendations for the standardization of criteria and use.

In this context, improving access to diagnosis, especially in Chagas-endemic areas, is crucial. Currently, diagnostic laboratories are scarce and often inadequately equipped and/or staffed. Additional priorities include updating and enhancing diagnostic algorithms and methods, as well as gaining a better understanding of whether additional field studies are needed to establish the potential of point-of-care strategies for the diagnosis of chronic Chagas disease.

Over the past 2 decades, commercial development has yielded rapid diagnostic tests (RDTs) designed for detecting *T*. *cruzi*-specific immunoglobulin G (IgG) [8,9]. RDTs are easy-to-use point-of-care diagnostic tools that typically provide results within 30 minutes. These devices do not require electrical equipment, are storable, and are generally functional at room temperature. Additionally, some can work with very small samples of whole blood, enabling diagnosis by using finger pricks (peripheral blood samples). However, it is important to note that, at present, RDTs are recommended only for screening purposes and not for individual diagnosis. Serological confirmation of a patient is mandatory, potentially causing a delay in initiating treatment when recommended [1].

Recent studies suggest using RDTs with whole-blood samples to provide a quick, reliable, and definitive individual diagnosis of Chagas disease in remote areas [10,11]. A systematic review and meta-analysis showed that, worldwide, the sensitivity of the RDTs examined was good (greater than 95%), with excellent specificity (>99%), regardless of their use in endemic or nonendemic regions [12]. Nonetheless, the results of the overall published literature vary [13,14].

The objective of the present discussion was to review the existing evidence supporting the use of RDTs for screening or diagnosing chronic Chagas disease. Additionally, the aim was to discuss the implications of eventually adopting and implementing these tests as a strategy for diagnosing Chagas disease, considering their current reported accuracy and sustainability as a public health response in Latin America.

## Methodology

PAHO, in partnership with Fiocruz, through the CUIDA Chagas project (Communities United for Innovation, Development and Attention for Chagas Disease—Toward elimination of congenital transmission of Chagas disease in Latin America), convened a group of Chagas disease experts in Salvador, Bahia, Brazil, from September 7 to 9, 2023. This meeting brought together a diverse panel of representatives from scientific and academic institutions from the Americas. Participation in the meeting was voluntary, and all the experts provided verbal informed consent for the use of the information provided.

The 3-day meeting facilitated discussions among more than 30 experts and participants from 9 countries (Argentina, Bolivia, Brazil, Chile, Colombia, Mexico, Uruguay, Switzerland, and the United States), addressing the objectives outlined earlier. The meeting agenda was structured around 3 main panels:

1. The use of RDTs for screening and diagnosis of chronic Chagas disease.
2. Existing research protocols for RDTs for Chagas disease.
3. Regulatory requirements and mechanisms for procuring RDTs for diagnosing Chagas disease in Latin American countries.

The nominal group technique, also known as the expert panel, was employed to gather firsthand information from relevant experts and determine the extent of their agreement on various topics [15]. Experts from the fields of clinical science, laboratory, parasitology, epidemiology, and research were purposefully sampled based on the diversity of their expertise and affiliations. Participation in the meeting was voluntary, and all the experts provided informed consent for the use of the information provided.

After each panel discussion, feedback was processed and summarized at key discussion points within the expert group. The meeting employed moderated plenary discussions. Deliberations took place in a total of 3 sessions of panel discussions in which the discussants offered their expert opinions. To complement the experts’ discussion, 3 questions were formulated and debated in groups of 7 to 8 participants. Post meeting, the summary reports from each group were collated to generate a plenary report and summary recommendations, which were revised and approved by all participants.

## Results

### Conclusions of the panel sessions

#### Session 1: The use of RDTs for screening and diagnosis of chronic Chagas disease

RDTs have not been the sole diagnostic test for patients with chronic Chagas disease, with limited studies exploring this possibility [10,16]. According to the consensus among experts, RDT should not be employed in the diagnosis of acute cases of Chagas disease or in blood donors [5]. Current application of RDTs for chronic Chagas disease is restricted to specific situations, like epidemiological surveys or screening initiatives. The suggested systematic use of RDTs is proposed for special circumstances, emergencies and among at-risk populations residing in remote areas where small, low-capacity laboratories are prevalent or absent. Additionally, these tests can also be considered potential second diagnostic tools for case confirmation when no other recommended laboratory techniques are available, with an understanding of the limitations of this approach.

The expert group called attention to the fact that RDTs offer multiple options, but few quality studies have evaluated their performance. The literature consistently shows high specificity but insufficient sensitivity, with varying results depending on the test used [17]. More than 90% of RDTs exhibit specificity exceeding 95%. However, there is significant variability in sensitivity, a crucial factor in RDT usage. Consequently, their application is termed a “diagnostic approximation” toward reaching a definitive diagnosis. However, further studies on this subject are imperative.

While RDTs are not utilized for treatment decisions, evidence suggests that they facilitate and potentially enhance access to diagnosis [18]. Due to their immediacy, a positive RDT result ensures prompt recruitment of patients with a suspected case for further study, confirmation, case management, potential referral, and/or treatment.

The group emphasized key advantages of RDTs, including portability, minimal blood sample and reagent volume, centrifugation-free processing, tolerance to temperature fluctuations (generally between 15 and 30 °C), and rapid results, facilitating swift and effective decision-making in specific situations. Additional advantages include ease of transport without refrigeration (except in higher temperature areas of Latin America) and applicability in non-laboratory settings.

Current RDTs detect specific antibodies using antigens selected by each manufacturer, necessitating evaluation by country-specific authorities. Notably, limited information exists about the trajectory, experience, and accumulated knowledge regarding the widespread use of RDTs in Chagas disease.

In general, RDTs exhibit excellent detection performance in patients with high antibody concentrations. However, challenges in effectiveness and performance may arise in Chagas disease cases with low antibody concentrations, representing 5% of the positive population, as presented in a study during the meeting [19]. Regarding RDT sample holders, experts stressed the critical need for permanent diagnostic test strips for subsequent verification and confirmation of readings, given that RDTs readings are operator dependent and that the strips may fade after a few minutes after use. The potential integration of smartphones-related methods to capture and document the output of test strips before they fade could be an option [20]. Additionally, practical constraints hinder simultaneous processing of large sample quantities, potentially resulting in delays in routine service delivery.

While some studies have assessed the performance of RDTs in Chagas disease [9], additional research is imperative to generate evidence that compares the effectiveness of available RDTs. Ideally, these studies should be conducted by research institutions and encompass diverse patient types and populations. Furthermore, there is currently insufficient evidence regarding the quality of RDTs for routine use, especially in primary care systems.

Comparative studies utilizing verified and accessible serum sample panels are essential for establishing standard procedural protocols. Strategies and criteria for the appropriate use of RDTs in various situations, including standard verification protocols, need to be developed, and evidence supporting their use must be established.

In conclusion, experts unanimously advocate that discussions on the future development of RDTs should be guided by the actual diagnostic needs of national health systems, patients, and users in general. The most effective RDTs will be those that align with real diagnostic needs or objectives set by providers of public health services.

#### Session 2: Use, applications, studies, and protocols on RDTs for Chagas disease

From 2003 to 2022, over 14 studies have explored various facets of Chagas disease diagnosis using RDTs [18]. These investigations covered diverse topics, including immunochromatography of sera from multiple origins, RDTs use in blood donors compared ELISA, and the field performance of RDTs in different demographic conditions, such as children, adolescents, and pregnant women, in both endemic and nonendemic regions. The studies also explored the use of RDTs among immigrants residing outside of Latin America and detected and compared the results from different RDTs [21].

Migration has brought the disease outside of the endemic countries, where the transmission continues vertically and via blood and tissue/organ donations. Recent studies have shown the pivotal role that RDTs for Chagas disease play in nonendemic regions [22]. Moreover, advancements in RDT technology have improved their sensitivity and specificity, enhancing their reliability in detecting Chagas disease even in low-prevalence settings [23]. By enabling early diagnosis and treatment, RDTs significantly contribute to public health efforts aimed at controlling the spread of this neglected tropical disease beyond its traditional endemic boundaries.

Experts concur a notable gap in cost-effectiveness and cost–outcome studies and that addressing those deficiencies is crucial for ensuring the accessibility and sustainability of RDTs. It is imperative to scrutinize related factors, including the regularity of production and supply. Moreover, various diagnostic technique combinations in studies suggest a sensitivity rate of 95%, encouraging exploration of different approaches like “duo” RDTs, a single RDT combined with a single ELISA, or a single RDT combined with 2 ELISAs. To achieve this, rigorous studies with quality control measures are crucial, integrating protocols into generic frameworks.

Regarding specificity and sensitivity, experts urge consensus to establish universally accepted thresholds for RDT use in public health services. Systematic reviews with appropriate methodologies are needed to develop tables outlining the use of RDTs. These tables should summarize evidence derived from baseline pretest diagnostic probability, based on epidemiologic risk (high-risk, medium-risk, and low-risk populations). Additionally, there is a call for the development of new and improved studies to fortify the existing body of knowledge and facilitate evidence-based recommendations. It is proposed to create generic protocols, setting harmonized standards and procedures and quality control measures. For this, a critical step involves verifying RDT performance in diverse settings and among specific populations. Understanding the purpose and target populations for RDTs in Chagas disease patients is essential, and options for their use in specific scenarios, such as outbreaks or emergencies, should be explored, particularly for vulnerable groups such as children and women of childbearing age.

#### Session 3: Regulatory requirements and mechanisms for procuring RDTs to diagnose Chagas disease in Latin American countries

In the realm of RDTs for Chagas disease, the PAHO Strategic Fund stands as a vital technical cooperation mechanism for RDTs in Chagas disease, enhancing access and availability of diagnostic supplies [24]. Adhering to specific criteria, The Strategic Fund ensures the quality of the In Vitro Diagnostic (IVD) tests for member countries and employs tools to enhance demand planning and procurement processes.

A recommendation was made to update the list of IVD supplies for Chagas disease within the Strategic Fund. This update, supported by a group of experts, aims to facilitate access to validated diagnostic tests and expand the portfolio, providing countries with alternatives.

To assist countries in managing RDT supply, support includes identifying critical areas in the supply cycle, guiding demand planning, and implementing best practices for RDT kit procurement. Several barriers to the use of RDTs were identified, including health workers’ lack of confidence in their accuracy, high costs, market sustainability challenges, and insufficient availability due to registration obstacles in the local health registries of some countries.

Regulatory challenges and obstacles include variations in clinical verification between countries, hindering health registration. Heterogeneous regulatory requirements, nonrecognition of international approvals, irregular production, market availability issues, and high costs also pose challenges in certain countries.

Opportunities for improvement in access to and availability of RDTs for the diagnosis of Chagas disease (identification of *T*. *cruzi* immunological response) have been identified. These opportunities include mapping acquisitions and estimating demand by country, describing the RDT market, conducting price studies in countries of the Region, and advocating for the harmonization of country regulations applicable to these diagnostic tests.

## Conclusion of the debate of questions

### Question 1

**Based on published evidence or ongoing studies**, **is it possible to change current practices or international guidelines (such as those of PAHO) regarding the use of RDTs for screening and/or diagnosing chronic Chagas disease? What evidence is available for the use of combined RDTs as part of the diagnostic process (RDT-based diagnostic algorithms)? Is it possible to compare and use existing evidence generated by published or ongoing studies?**

The consensus reached by experts emphasizes the current inadequacy of evidence to advocate for a shift in recommended practices concerning the use of RDTs in diagnosing individuals with Chagas disease. However, a strong recommendation emerges for the endorsement and backing of RDTs as valuable tools for surveillance and screening purposes. The continued use of these tools will increase the body of knowledge and evidence supporting the potential expansion of RDT usage in the future.

To enhance the quality and comparability of future studies, the expert group advised the development and adoption of standardized generic protocols. This approach is particularly crucial in the context of field studies, where comprehensive documentation of surrounding conditions, such as geographical area, bioecology, population type, and prevalence, is paramount. It is equally important to document the role of genetic variations of *T*. *cruzi* (described as Discrete Typing Units [DTUs]). Such a recording is expected to contribute substantially and robustly to the accumulation of knowledge on the subject.

### Question 2

**Should RDTs be selected based solely on the sensitivity and specificity cutoff points indicated in the information published by the manufacturer? Or is it based on the performance of a certain brand over others? What other parameters or information**, **in addition to sensitivity and specificity, are essential for choosing an RDT for the diagnosis of chronic Chagas disease (considering endemicity and prevalence)?**

The consensus reached throughout the group’s discussion and plenary session concluded that the sensitivity and specificity reported by the manufacturer are insufficient for countries, public health systems and users to select a determined RDT. Verification of the sensitivity and specificity of RDTs is a critical responsibility that should rest with competent national authorities, including entities such as the national reference laboratory for public health. These authorities must ensure thorough documentation and reporting of the performance of RDTs within the country, disseminating this information through channels such as laboratory networks and end-users.

In addition to assessing sensitivity and specificity, it is crucial to document the positive predictive value of RDTs, evaluating their ease of use and practicality across diverse environments (urban versus rural, children versus adults). Furthermore, studies are necessary to examine the sustainability of the production and availability of existing RDTs. Additionally, conducting cost–benefit and cost-effectiveness studies is essential to provide a holistic understanding of their utility and impact.

### Question 3

**Is preliminary geographic validation of RDT performance necessary before using RDTs in a particular geographic region**, **especially for diagnosing chronic Chagas disease?**

The group unanimously confirmed the necessity of geographical validation of RDTs. The criteria for each country or subregion should be utilized for this purpose based on existing information regarding the similarity or diversity of bioecogeographic environments, genetic variations of the microorganism (DTUs), or the type of population to be screened. Emphasis was placed on the importance of distinguishing validation processes from verification processes. Manufacturers should conduct validation, including providing a comprehensive description of all RDT criteria and information.

On the other hand, verification should be performed by competent national authorities, such as the national reference laboratories of public health, using established protocols (for instance, Clinical and Laboratory Standards Institute (CLSI)). These protocols should require small sample sizes to evaluate RDT performance under more specific conditions, such as different batches of tests, ethnic groups, population types, or reactivity.

## Conclusions

Various options for RDTs exist, but the current evidence regarding their performance in diagnosing Chagas disease is both heterogeneous and insufficient. RDTs, however, hold significant potential in public health, particularly in areas with limited access to health services, emergencies, screening, and field surveys. Despite their potential, technological and operational challenges persist, encompassing issues such as sensitivity, interpretation of results, test selection, availability, accessibility, and cost.

The development and implementation of simple, rapid tests and devices for point-of-care screening and treatment of neglected tropical diseases have significantly reduced morbidity and mortality in regions with limited access to laboratory services [25]. For instance, RDTs for malaria and dengue have made accurate diagnoses more available and feasible, thereby improving the quality of care [26–28].

Recognizing the pressing need to address diverse problems and diagnostic scenarios, which demand comprehensive approaches, PAHO has proposed formulating a generic protocol. This protocol will facilitate the development of multicenter national or regional studies that assess RDT performance, effectiveness, and costs. To support this initiative, a yet-to-be-established working group is recommended that is tasked with preparing a target product profile. This profile could guide the development and testing of RDTs, aiming to position them as screening tools and/or for complementary diagnosis of Chagas disease across different countries of the Region of the Americas.

## References

[1] World Health Organization. Chagas disease (American trypanosomiasis) [Internet]. 2016 [cited 2023 Jun 14]. https://www.who.int/news-room/fact-sheets/detail/chagas-disease-(american-trypanosomiasis)

[2] de Sousa AS, Vermeij D, Ramos AN, Luquetti AO. Chagas disease. Lancet [Internet]. 2023 Dec [cited 2023 Dec 12]. https://pubmed.ncbi.nlm.nih.gov/38071985/

[3] Costa Chaves G, Abi-Saab Arrieche M, Rode J, Mechali D, Ouverney Reis P, Vieira Alves R, et al. Estimación de la demanda de medicamentos antichagásicos: una contribución para el acceso en América Latina. Rev Panam Salud Publica [Internet]. 2017 [cited 2023 Jun 14]. https://iris.paho.org/handle/10665.2/33999 10.26633/RPSP.2017.45 PMC6612731 28614468

[4] Pan American Health Organization. Guidelines for the Diagnosis and Treatment of Chagas Disease [Internet]. 2019. https://iris.paho.org/bitstream/handle/10665.2/49653/9789275120439_eng.pdf

[5] Picado A, Angheben A, Marchiol A, Alarcón de Noya B, Flevaud L, Pinazo MJ, et al. Development of Diagnostics for Chagas Disease: Where Should We Put Our Limited Resources? PLoS Negl Trop Dis [Internet]. 2017 Jan 5 [cited 2023 Jun 14];11(1):e0005148. Available from: https://journals.plos.org/plosntds/article?id=10.1371/journal.pntd.0005148 28056025 10.1371/journal.pntd.0005148 PMC5221646

[6] Balouz V, Agüero F, Buscaglia CA. Chagas Disease Diagnostic Applications: Present Knowledge and Future Steps. Adv Parasitol [Internet]. 2017 [cited 2023 Jun 14];97:1–45. Available from: https://pubmed.ncbi.nlm.nih.gov/28325368/ 28325368 10.1016/bs.apar.2016.10.001 PMC5363286

[7] Schijman AG. Molecular diagnosis of Trypanosoma cruzi. Acta Trop [Internet]. 2018 Aug 1 [cited 2023 Jul 11];184:59–66. Available from: https://pubmed.ncbi.nlm.nih.gov/29476727/ 29476727 10.1016/j.actatropica.2018.02.019

[8] Junqueira ÂCV. International meeting: new diagnostic tests are urgently needed to treat patients with Chagas disease. Rev Soc Bras Med Trop [Internet]. 2008 May [cited 2023 Jun 14];41(3):315–319. Available from: https://pubmed.ncbi.nlm.nih.gov/18719818/ 18719818 10.1590/s0037-86822008000300020

[9] Sánchez-Camargo CL, Albajar-Viñas P, Wilkins PP, Nieto J, Leiby DA, Paris L, et al. Comparative evaluation of 11 commercialized rapid diagnostic tests for detecting Trypanosoma cruzi antibodies in serum banks in areas of endemicity and nonendemicity. J Clin Microbiol [Internet]. 2014 [cited 2023 Jun 14];52(7):2506–2512. Available from: https://pubmed.ncbi.nlm.nih.gov/24808239/ 24808239 10.1128/JCM.00144-14 PMC4097696

[10] Egüez KE, Alonso-Padilla J, Terán C, Chipana Z, García W, Torrico F, et al. Rapid diagnostic tests duo as alternative to conventional serological assays for conclusive Chagas disease diagnosis. PLoS Negl Trop Dis [Internet]. 2017 Apr 3 [cited 2023 Jun 14];11(4):e0005501. Available from: https://journals.plos.org/plosntds/article?id=10.1371/journal.pntd.0005501 28369081 10.1371/journal.pntd.0005501 PMC5391121

[11] Lozano D, Rojas L, Méndez S, Casellas A, Sanz S, Ortiz L, et al. Use of rapid diagnostic tests (RDTs) for conclusive diagnosis of chronic Chagas disease–field implementation in the Bolivian Chaco region. PLoS Negl Trop Dis [Internet]. 2019 Dec 19 [cited 2023 Jun 14];13(12):e0007877. Available from: https://journals.plos.org/plosntds/article?id=10.1371/journal.pntd.0007877 31856247 10.1371/journal.pntd.0007877 PMC6922313

[12] Angheben A, Buonfrate D, Cruciani M, Jackson Y, Alonso-Padilla J, Gascon J, et al. Rapid immunochromatographic tests for the diagnosis of chronic Chagas disease in at-risk populations: A systematic review and meta-analysis. PLoS Negl Trop Dis [Internet]. 2019 May 1 [cited 2023 Jun 14];13(5):e0007271. Available from: https://journals.plos.org/plosntds/article?id=10.1371/journal.pntd.0007271 31150377 10.1371/journal.pntd.0007271 PMC6561601

[13] Marchiol A, Sanchez ACF, Caicedo A, Segura M, Bautista J, Sotelo MSA, et al. Laboratory evaluation of eleven rapid diagnostic tests for serological diagnosis of Chagas disease in Colombia. PLoS Negl Trop Dis [Internet]. 2023 Aug 1 [cited 2024 May 20];17(8):e0011547. Available from: https://journals.plos.org/plosntds/article?id=10.1371/journal.pntd.0011547 37607214 10.1371/journal.pntd.0011547 PMC10473487

[14] Truyens C, Dumonteil E, Alger J, Cafferata ML, Ciganda A, Gibbons L, et al. Geographic Variations in Test Reactivity for the Serological Diagnosis of Trypanosoma cruzi Infection. J Clin Microbiol [Internet]. 2021 Nov 1 [cited 2024 May 20];59(12). Available from: https://pubmed.ncbi.nlm.nih.gov/34469183/ 34469183 10.1128/JCM.01062-21 PMC8601237

[15] Jones J, Hunter D. Consensus methods for medical and health services research. BMJ [Internet]. 1995 Aug 5 [cited 2024 Jan 30];311(7001):376. Available from: https://pubmed.ncbi.nlm.nih.gov/7640549/ 7640549 10.1136/bmj.311.7001.376 PMC2550437

[16] Ortega-Arroyo A, Flores-Chavez MD, Puente-Alcaraz J. Combined use of two rapid tests for the conclusive diagnosis of Chagas disease: a systematic scoping review. BMJ Open [Internet]. 2021 Oct 29 [cited 2024 Jan 30];11(10). Available from: https://pubmed.ncbi.nlm.nih.gov/34716159/ 34716159 10.1136/bmjopen-2020-047825 PMC8559098

[17] Schijman AG, Alonso-Padilla J, Longhi SA, Picado A. Parasitological, serological and molecular diagnosis of acute and chronic Chagas disease: from field to laboratory. Mem Inst Oswaldo Cruz [Internet]. 2022 [cited 2024 Jan 30];117(1). Available from: https://pubmed.ncbi.nlm.nih.gov/35613155/10.1590/0074-02760200444 PMC9164950 35613155

[18] Gabaldón-Figueira JC, Skjefte M, Longhi S, Escabia E, García LJ, Ros-Lucas A, et al. Practical diagnostic algorithms for Chagas disease: a focus on low resource settings. Expert Rev Anti Infect Ther [Internet]. 2023 [cited 2024 Jan 30];21(12):1287–1299. Available from: https://pubmed.ncbi.nlm.nih.gov/37933443/ 37933443 10.1080/14787210.2023.2279110

[19] Lopez-Albizu C, Rivero R, Ballering G, Freilij H, Santini MS, Bisio MMC. Laboratory diagnosis of Trypanosoma cruzi infection: a narrative review. Front Parasitol. 2023 May 24;2:1138375. 10.3389/fpara.2023.1138375 PMC11732150 39816836

[20] Wood CS, Thomas MR, Budd J, Mashamba-Thompson TP, Herbst K, Pillay D, et al. Taking connected mobile-health diagnostics of infectious diseases to the field. Nature [Internet]. 2019 Feb 28 [cited 2024 May 20];566(7745):467–474. Available from: https://pubmed.ncbi.nlm.nih.gov/30814711/ 30814711 10.1038/s41586-019-0956-2 PMC6776470

[21] Higuita NIA, Franco-Paredes C, Henao-Martínez AF, Beatty NL, Manne-Goehler J, Forsyth CJ. Chagas Disease and Domestic Medical Screening Guidance for Newly Arrived Individuals Under a Humanitarian-Based Immigration Status: A Call for Action. Am J Trop Med Hyg [Internet]. 2022 Nov 1 [cited 2024 Jan 30];107(5):960–963. Available from: https://pubmed.ncbi.nlm.nih.gov/36395747/ 36395747 10.4269/ajtmh.22-0309 PMC9709006

[22] Elkheir N, Carter J, García-Mingo A, Chiodini P. Chagas disease in non-endemic settings. BMJ [Internet]. 2021 Apr 9 [cited 2024 May 20];373. Available from: https://pubmed.ncbi.nlm.nih.gov/33837041/ 33837041 10.1136/bmj.n901

[23] Chappuis F, Mauris A, Holst M, Albajar-Vinas P, Jannin J, Luquetti AO, et al. Validation of a Rapid Immunochromatographic Assay for Diagnosis of Trypanosoma cruzi Infection among Latin-American Migrants in Geneva, Switzerland. J Clin Microbiol [Internet]. 2010 Aug [cited 2024 May 20];48(8):2948. Available from: https://www.ncbi.nlm.nih.gov/pmc/articles/PMC2916554/ 20554821 10.1128/JCM.00774-10 PMC2916554

[24] PAHO Strategic Fund—PAHO/WHO | Pan American Health Organization [Internet]. [cited 2024 Jan 30]. https://www.paho.org/en/paho-strategic-fund

[25] Barbé B, Verdonck K, El-Safi S, Khanal B, Teav S, Lilo Kalo JR, et al. Rapid Diagnostic Tests for Neglected Infectious Diseases: Case Study Highlights Need for Customer Awareness and Postmarket Surveillance. PLoS Negl Trop Dis [Internet]. 2016 Nov 3 [cited 2024 May 20];10(11). Available from: https://pubmed.ncbi.nlm.nih.gov/27812099/ 27812099 10.1371/journal.pntd.0004655 PMC5094593

[26] Kavanaugh MJ, Azzam SE, Rockabrand DM. Malaria Rapid Diagnostic Tests: Literary Review and Recommendation for a Quality Assurance, Quality Control Algorithm. Diagnostics (Basel) [Internet]. 2021 [cited 2024 May 20];11(5). Available from: https://pubmed.ncbi.nlm.nih.gov/33922917/ 33922917 10.3390/diagnostics11050768 PMC8145891

[27] Ndiaye O, Woolston K, Gaye A, Loucoubar C, Cocozza M, Fall C, et al. Laboratory Evaluation and Field Testing of Dengue NS1 and IgM/IgG Rapid Diagnostic Tests in an Epidemic Context in Senegal. Viruses [Internet]. 2023 Mar 31 [cited 2024 May 20];15(4):904–904. Available from: https://europepmc.org/articles/PMC10143717 37112887 10.3390/v15040904 PMC10143717

[28] Pollak NM, Olsson M, Ahmed M, Tan J, Lim G, Setoh YX, et al. Rapid Diagnostic Tests for the Detection of the Four Dengue Virus Serotypes in Clinically Relevant Matrices. Microbiol Spectr [Internet]. 2023 Feb 14 [cited 2024 May 20];11(1). Available from: https://pubmed.ncbi.nlm.nih.gov/36682882/ 36682882 10.1128/spectrum.02796-22 PMC9927141

